# An arylsulfonamide that targets cell wall biosynthesis in *Mycobacterium tuberculosis*

**DOI:** 10.1128/aac.01037-24

**Published:** 2024-09-26

**Authors:** Renee Allen, Lauren Ames, Vanessa Pietrowski Baldin, Arielle Butts, Kenneth J. Henry, Greg Durst, Diana Quach, Joseph Sugie, Joe Pogliano, Tanya Parish

**Affiliations:** 1 Center for Global Infectious Disease Research, Seattle Children’s Research Institute, Seattle, Washington, USA; 2 Lgenia, Fortville, Indiana, USA; 3 Linnaeus Bioscience Inc., San Diego, California, USA; 4 Department of Pediatrics, University of Washington School of Medicine, Seattle, Washington, USA; St. George's, University of London, London, United Kingdom

**Keywords:** tuberculosis, mycobacteria, cell wall, MmpL3, antitubercular, mode of action

## Abstract

We investigated the mechanism of action of an arylsulfonamide with whole-cell activity against *Mycobacterium tuberculosis*. We newly synthesized the molecule and confirmed it had activity against both extracellular and intracellular bacilli. The molecule had some activity against HepG2 cells but maintained some selectivity. Bacterial cytological profiling suggested that the mechanism of action was via disruption of cell wall synthesis, with similarities to an inhibitor of the mycolic acid exporter MmpL3. The compound induced expression from the IniB promoter and caused a boost in ATP production but did not induce reactive oxygen species. A mutation in MmpL3 (S591I) led to low-level resistance. Taken together, these data confirm the molecule targets cell wall biosynthesis with MmpL3 as the most probable target.

## INTRODUCTION

Infections caused by *Mycobacterium tuberculosis* still remain a serious global health problem, with >1 million deaths every year (1). In order to improve drug treatment and overcome resistance, the pipeline of new drugs needs to be constantly refilled. Phenotypic screening has identified many new drug classes with potential for development. Identification of the bacterial target for whole-cell active compounds is an important component of hit evaluation and progression.

We are interested in identifying the targets of molecules identified in whole-cell screens. An arylsulfonamide was identified by screening the AstraZeneca corporate library (320,000 compounds), and limited structure–activity relationship studies were conducted (2). The series appeared to have a novel target since it did not inhibit mycolic acid synthesis (InhA), oxidative phosphorylation, DNA replication, transcription, or translation, and the target was therefore uncharacterized (2).

TPN-0157345 (2-methyl-4-(1*H*-pyrazol-1-yl)- *N*-(1-(pyridin-4-ylmethyl) piperidin-4-yl) benzenesulfonamide) was prepared according to the literature procedure (2). We found that the inhibitory concentration (IC_90_) against aerobically cultured *M. tuberculosis* H37Rv was 3.3 µM, which is consistent with the previously reported minimum inhibitory concentration (MIC) of 1.6 µM^2^ (Table 1). We also tested the molecule for activity against intracellular *M. tuberculosis* H37Rv in THP-1 macrophages and found that the IC_90_ was 1.4 µM, again consistent with the previous report that the molecule had activity against bacteria inside THP-1 cells, although an MIC was not determined previously (2). We determined cytotoxicity against the HepG2 liver cell line; the IC_50_ was 22 µM, giving a selectivity index of ~11. Previous work tested cytotoxicity against THP-1 cells, which was very similar at 32 µM^2^.

**TABLE 1:** Compound activity

	IC_50_ (µM)	IC_90_ (µM)	Fold change
Extracellular activity (wild-type)	2.1 ± 0.2	3.3 ± 1.3	
Intracellular activity	0.5 ± 0.06	1.4 ± 0.7	
HepG2 cytotoxicity	22 ± 4.2	ND	
Extracellular activity (MmpL3 F255L, V646M, F644I)	1.4 ± 0.8	5.6 ± 5.3	1.7
Extracellular activity (MmpL3 S591I)	4.1 ± 0.7	9.2 ± 2.5	2.8

Activity against *M. tuberculosis* strains (all derived from H37Rv London Pride [ATCC 25618]) was measured in Middlebrook 7H9 medium with 10% v/v Middlebrook OADC supplement and 0.05% w/v Tween 80 (3) or in THP-1-infected cells (multiplicity of infection of 1) (4). Cytotoxicity was measured against HepG2 cells (4). Data are the average and standard deviation of two independent experiments. Fold change is between wild-type and MmpL3 mutant strains (IC_90_).

**FIG 1:**
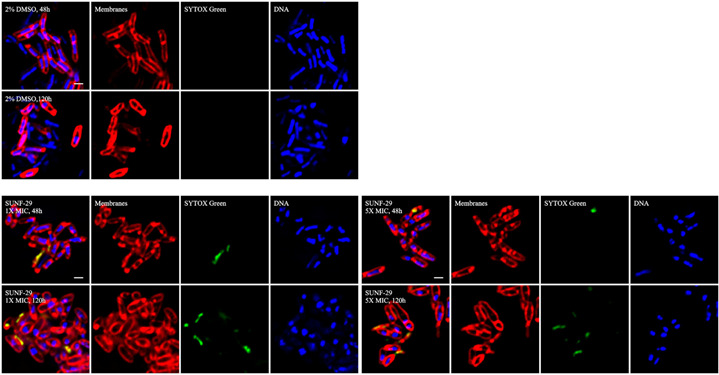
Bacterial cytological profiling. *M. tuberculosis* mc^2^6206 was inoculated into medium at an OD of 0.06 to 0.08, treated with compounds for 48–120 h, and fixed with 16% paraformaldehyde, 8% glutaraldehyde, and 0.4 M phosphate buffer (pH 7.5). Cells were washed twice with a warm medium and stained for 30 min with FM 4-64 (Invitrogen), SYTO 40 (Invitrogen), and SYTOX Green (Invitrogen). Membranes are imaged in red, DNA in blue, and green staining indicates that cell integrity has been compromised; scale bars are 1 µm.

Once we had confirmed that the newly synthesized molecule was active and retained similar properties to the previous report, we conducted bacterial cytological profiling (BCP) to identify the pathway(s) that are inhibited by this molecule (5). BCP uses changes in cellular morphology effected by compound exposure to classify target pathways. *M. tuberculosis* mc^2^6206 was exposed to compounds for 48 and 120 h at two concentrations (Fig. 1). The bacterial cells became rounded or ovoidal with increased membrane permeability at both concentrations. Comparison with a database of control molecules revealed the closest similarity to bacteria treated with a known MmpL3 inhibitor, the adamantyl urea AU1235, suggesting that the compounds affect the cell wall.

**FIG 2:**
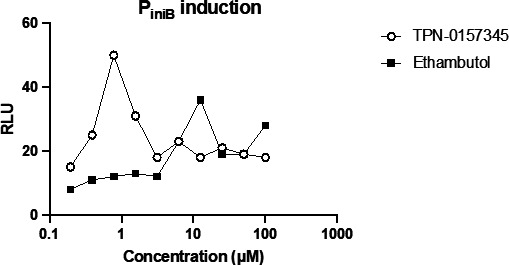
Exposure to the arylsulfonamide induces P_iniB_ in *M. tuberculosis*. Recombinant *M. tuberculosis* expressing Lux under the control of the IniBAC promoter (P_iniB_-Lux)^6^ was cultured to logarithmic phase and exposed to compounds for 3 days. Luciferase activity was measured after the addition of luciferin by measuring relative luminescence units (RLU). Data are representative of two fully independent runs (see Fig. S1).

Our data suggested that the molecule targets cell wall synthesis. Increased expression of the IniBAC operon is observed with inhibitors of cell wall synthesis, including MmpL3 inhibitors (6). Therefore, we looked at the induction of the IniB promoter using a reporter strain of *M. tuberculosis* (Fig. 2). We saw a clear concentration-dependent increase in expression from P_iniB_ at concentrations around the MIC (at higher concentrations, the induction is lost, presumably due to cell death). Similarly, the positive control, ethambutol, led to increased expression from P_iniB_ in a concentration-dependent manner.

**FIG 3:**
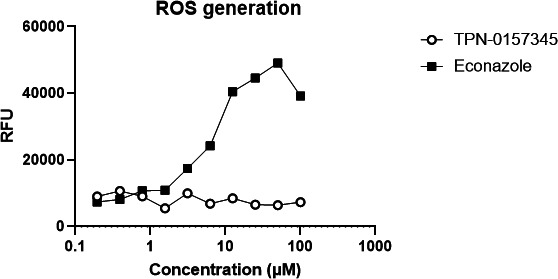
Exposure to the arylsulfonamide does not induce the production of reactive oxygen species (ROS) in *M. tuberculosis. M. tuberculosis* (H37Rv) was cultured to the logarithmic phase and loaded with 40 µM dichlorodihydrofluorescein diacetate. Cultures were exposed to compounds for 90 min, and the production of ROS was determined by measuring relative fluorescence units (RFU) at Ex485/Em535. Data are representative of two fully independent runs (see Fig. S1).

We tested whether compounds induced general stress by determining whether there was any generation of reactive oxygen species (ROS) (Fig. 3). We used the fluorescent reporter dye dichlorodihydro-fluorescein diacetate to monitor ROS. No generation of ROS was seen after exposure to the arylsulfonamide compound, confirming that this is not the mechanism of action. The positive control, econazole, induced ROS in a concentration-dependent manner.

**FIG 4:**
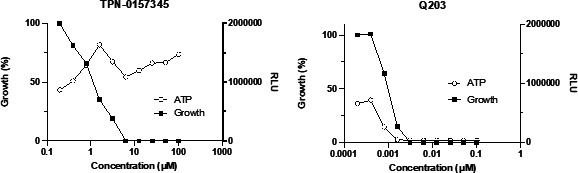
Exposure to the arylsulfonamide leads to a boost in ATP production in *M. tuberculosis. M. tuberculosis* (H37Rv) was cultured to logarithmic phase and exposed to compounds for 24 h. ATP was measured using the BacTiter Glo reagent after incubation for 10 min in the dark and measuring relative luminescence units (RLU). Growth was determined after 5 days by reading OD_590_ and normalized to the dimethylsulfoxide (DMSO) control. Data are representative of two fully independent runs (see Fig. S1).

Previous work has shown that cell wall inhibitors can lead to a boost in ATP production in *Mycobacterium bovis* BCG (7). We tested the effect of the compound on ATP levels. We saw an increase in ATP levels at concentrations around the MIC (Fig. 4). ATP levels remained high even when bacterial growth was inhibited, demonstrating increased ATP production consistent with targeting cell wall biosynthesis. As expected, the positive control Q203, which inhibits the electron transport chain, resulted in ATP depletion at concentrations below the MIC.

MmpL3 is a promiscuous target and can be inhibited by many chemical scaffolds (8). We tested whether the compounds were active against *M. tuberculosis* strains carrying mutations in MmpL3. We first tested a strain with three MmpL3 mutations (F255L, V646M, F644I) (9), which are resistant to multiple chemical scaffolds, but found no difference in MIC (Table 1). However, when testing a strain with a different (single) mutation (S591I), we saw a shift toward low-level resistance. These data are consistent with the molecule targeting MmpL3 but indicate that its mode of binding may be different from several chemical classes such as the spiral amines and adamantly ureas (9).

**FIG 5:**
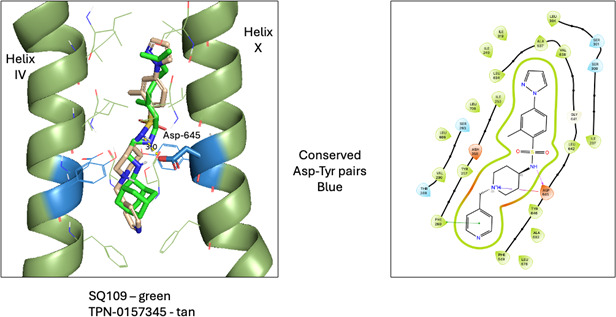
Molecular docking and interaction diagram of TPN-0157345 in *M. smegmatis* MmpL3 (PDB accession number 6AJG).

In order to support the hypothesis that TPN-0157345 targets MmpL3, we undertook a molecular docking study to judge the likelihood that the compound can bind this target, and to compare the three-dimensional (3D) structure with that of a known inhibitor. Molecular docking was performed with Glide (Schrödinger Release 2023-3) using the structure of *Mycobacterium smegmatis* MmpL3 in complex with SQ109 (PDB: 6AJG) (10). The protein and ligand were prepared with Maestro, docked with Glide, and visualized with PyMOL v2.5.0 (Schrödinger). Many MmpL3 ligands have a common hydrogen bond donor that interacts with Asp-645 that is part of two conserved Asp-Tyr pairs located in the essential proton translocation region (10, 11). TPN-0157345 has two hydrogen bond donors, an amide NH and a basic (likely protonated) piperidine. The docking produced a pose, with the amide NH directly interacting with Asp-645 (Fig. 5). The piperidine NH+ is within interaction distance to the Asp for a charged species. The fact that this docking pose identified critical interactions between the inhibitor and the protein that are common to other MmpL3 inhibitors and the very high 3D similarity with SQ109 strongly supports the assignment of MmpL3 as the target of this compound.

In summary, exposure to the arylsulfonamide molecule inhibits cell wall synthesis and induces an ATP boost. Mutation in MmpL3 confers resistance to the molecule. Our data are consistent with a mode of action in which the arylsulfonamide targets MmpL3.

## SUPPLEMENTAL MATERIAL

Figure S1 aac.01037-24-s0001.pdf (A) Induction of P_iniB_, (B) production of ROS, and (C) ATP boost in *M. tuberculosis*.

ASM does not own the copyrights to Supplemental Material that may be linked to, or accessed through, an article. The authors have granted ASM a non-exclusive, world-wide license to publish the Supplemental Material files. Please contact the corresponding author directly for reuse.
