# Supplementary material for: An arylsulfonamide that targets cell wall biosynthesis in Mycobacterium tuberculosis
Source: Antimicrob Agents Chemother. 2024 Sep 26;68(11):e01037-24. doi: 10.1128/aac.01037-24 (PMC11539219; doi:10.1128/aac.01037-24)
Supplement: Figure S1 — (A) Induction of PiniB, (B) production of ROS, and (C) ATP boost in M. tuberculosis. [file aac.01037-24-s0001.pdf]

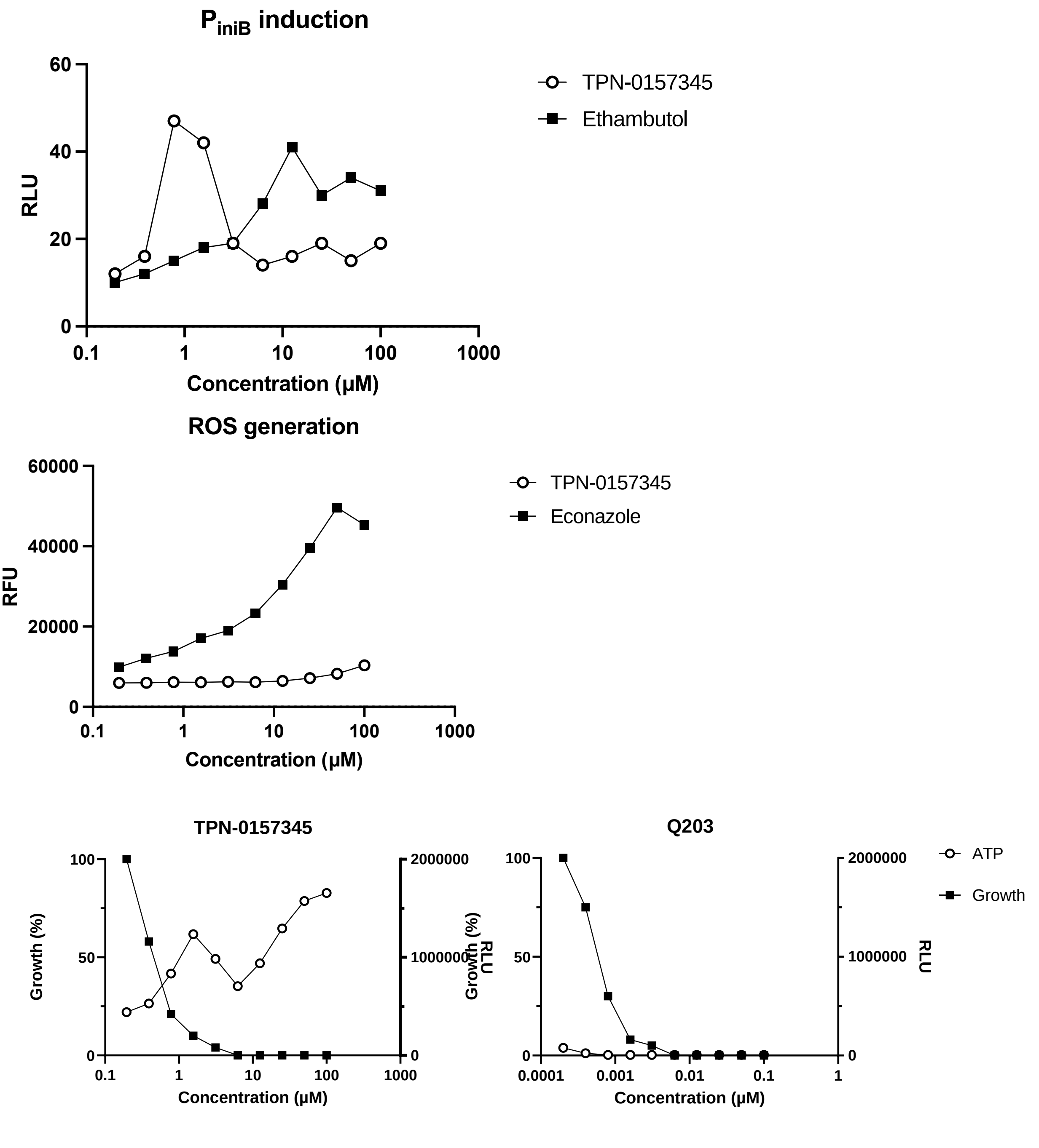

Figure S1. A. Induction of PiniB in *M. tuberculosis*. Recombinant *M. tuberculosis* expressing Lux under the control of the IniBAC promoter (P_{iniB}-Lux) was cultured to logarithmic phase and exposed to compounds for 3 days. Luciferase activity was measured after the addition of luciferin by measuring relative luminescence units (RLU). **B. Production of ROS in *M. tuberculosis*.** *M. tuberculosis* (H37Rv) was cultured to logarithmic phase and loaded with 40 µM dichlorodihydrofluorescein diacetate. Cultures were exposed to compounds for 90 min and the production of ROS was determined by measuring relative fluorescence units (RFU) at Ex485/Em535. **C. ATP boost in *M. tuberculosis*.** *M. tuberculosis* (H37Rv) was cultured to logarithmic phase and exposed to compounds for 24 h. ATP was measured using the BacTiter Glo reagent after incubation for 10 min in the dark and measuring RLU. Growth was determined after 5 d by reading OD_{590} and normalized to the DMSO control
